# Cleft Palate and Hare Lip

**Published:** 1906-01

**Authors:** 


Cleft Palate and Hare Lip. By W. Arbuthnot Lane, M.S., F. R. C. S., Surgeon to Guy's Hospital and Senior Surgeon to the Hospital for Sick Children, Great Ormond St., London. Medical Publishing Co., (Limited).

This monograph is issued in quarto form, and as the British *Medical Journal* says, it is really a "sight for sore eyes," so large and legible is the type. The illustrations are equally as well executed. The subject-matter consists of a *résume* of the various articles the author has written from time to time on Cleft Palate and Hare Lip. This includes all the important features concerning the operation as well as a discussion in detail of the factors that influence the growth of the naso-pharynx and mouth. This last point is of much importance " since the success which attends these operations, so far as concerns perfection of speech, varies directly with the development of the naso-pharynx and with the freedom of the passage of air through it."

